# Functional connectivity varies across scales in a fragmented landscape

**DOI:** 10.1371/journal.pone.0289706

**Published:** 2023-08-09

**Authors:** Kate I. T. Bird, Daniel R. Uden, Craig R. Allen

**Affiliations:** 1 Center for Resilience in Agricultural Working Landscapes, School of Natural Resources, University of Nebraska-Lincoln, Lincoln, Nebraska, United States of America; 2 Department of Agronomy and Horticulture, University of Nebraska-Lincoln, Lincoln, Nebraska, United States of America; The University of Auckland - City Campus: University of Auckland, NEW ZEALAND

## Abstract

Species of different sizes interact with the landscape differently because ecological structure varies with scale, as do species movement capabilities and habitat requirements. As such, landscape connectivity is dependent upon the scale at which an animal interacts with its environment. Analyses of landscape connectivity must incorporate ecologically relevant scales to address scale-specific differences. Many evaluations of landscape connectivity utilize incrementally increasing buffer distances or other arbitrary spatial delineations as scales of analysis. Instead, we used a mammalian body mass discontinuity analysis to objectively identify scales in the Central Platte River Valley (CPRV) of Nebraska, U.S.A. We implemented a graph-theoretic network analysis to evaluate the connectivity of two wetland land cover types in the CPRV, wet meadow and emergent marsh, at multiple scales represented by groupings of species with similar body mass. Body mass is allometric with multiple traits of species, including dispersal distances. The landscape was highly connected at larger scales but relatively unconnected at smaller scales. We identified a threshold at which the landscape becomes highly connected between 500 m and 6,500 m dispersal distances. The presence of a connectivity threshold suggests that species with dispersal distances close to the threshold may be most vulnerable to habitat loss or reconfiguration and management should account for the connectivity threshold. Furthermore, we propose that a multiscale approach to management will be necessary to ensure landscape connectivity for diverse species.

## Introduction

Human-driven disturbances such as land use change produce scale-specific effects and responses in ecosystems [1]. At each spatiotemporal scale, different biotic and abiotic processes structure ecosystems, creating a scale-dependent suite of responses [1–3]. These processes and the resulting ecosystem structure also entrain attributes of animals, including how animals perceive and exploit the landscape [4]. Species may co-exist in the same geographic area but experience and move through the landscape differently because scale of interaction determines resource availability, habitat requirements, and species movement capabilities [1, 4, 5]. An understanding of how species at different scales perceive and interact with a given landscape will help anticipate the effects of future disturbances and inform ecosystem management and conservation efforts [1, 5, 6]. Multiscale approaches to management and conservation that incorporate a range of species are necessary for preventing the loss of biodiversity and maintaining resilient ecosystems [3]. Investigating patterns of connectivity for ecological communities, and how these patterns change with scale, will increase the likelihood of successful ecosystem management.

Landscape connectivity is species- and scale-dependent. Connectivity may describe (1) structural connectivity, or the spatial arrangement of habitat patches, and (2) functional connectivity, or how species move through the landscape [7–9]. For instance, in a fragmented landscape, species that interact with their environment at a larger scale will experience a more connected landscape than species at smaller scales because they possess a greater capability to move between distant habitat patches [6, 10, 11]. Previous studies have examined the influence of scale on connectivity and, for example, identified thresholds of connectivity that represent the minimum species dispersal distance at which the landscape is connected [e.g., 6, 8, 12–15]. Knowledge of how scale affects landscape connectivity is critical for ensuring that management efforts such as habitat conservation and restoration benefit the intended species in an ecosystem.

Scales are frequently assigned arbitrarily or are applicable to only a single species or subset of species, limiting the utility of any results and raising the possibility that the selected scales are irrelevant for the processes or species of focus [1, 16, 17]. Scales of management must align with or transcend multiple ecologically relevant scales to maximize beneficial outcomes for ecological communities, given that communities consist of multiple species interacting with the landscape at different scales [1, 5, 18, 19]. This may be especially challenging when the dispersal abilities of species within a community differ substantially because it requires evaluating connectivity across a large range of dispersal distances (i.e., extent) at a sufficient incremental resolution (i.e., grain). Discontinuity theory presents a method to objectively identify scales in a variety of systems, including ecological systems [1, 17, 20, 21]. Discontinuity theory emerged from Holling’s [4] conception of ecosystems, in which the organization of ecosystems sets a template for the structure of their animal communities, specifically body mass distributions. This approach identifies aggregations of species, which represent the species at a given scale of the ecosystem. Breaks between aggregations of species in the body mass distribution separate scales, indicating discontinuities in the ecological processes that structure the system [4]. Body mass discontinuity analyses have previously been applied to identify scales in studies of biological invasion and extinction [22, 23] and population variability [24], among others [e.g., 25, 26]. However, discontinuity analysis has not previously been utilized as a method to identify ecological scales in the context of landscape connectivity.

We applied a body mass discontinuity analysis to identify ecological scales for the analysis of landscape connectivity for mammals in the Central Platte River Valley (CPRV) in Nebraska, U.S.A. Body mass is allometric with multiple traits of species, including mammalian dispersal distances [27]. We utilized a mammalian body mass discontinuity analysis to identify scales in the ecosystem represented by groupings of species with similar body mass. To serve as an example of multiscale analysis of connectivity, we implemented a graph-theoretic network analysis to evaluate the connectivity of two wetland land cover types, wet meadow and emergent marsh, at the identified scales in the CPRV. We evaluated how node-level and landscape-level connectivity metrics vary across scales and identified thresholds of connectivity in the landscape. We also examined the utility of body mass discontinuity analysis as a method for objectively identifying ecological scales in analyses of landscape connectivity.

## Materials and methods

### Study area and data

**Fig 1:**
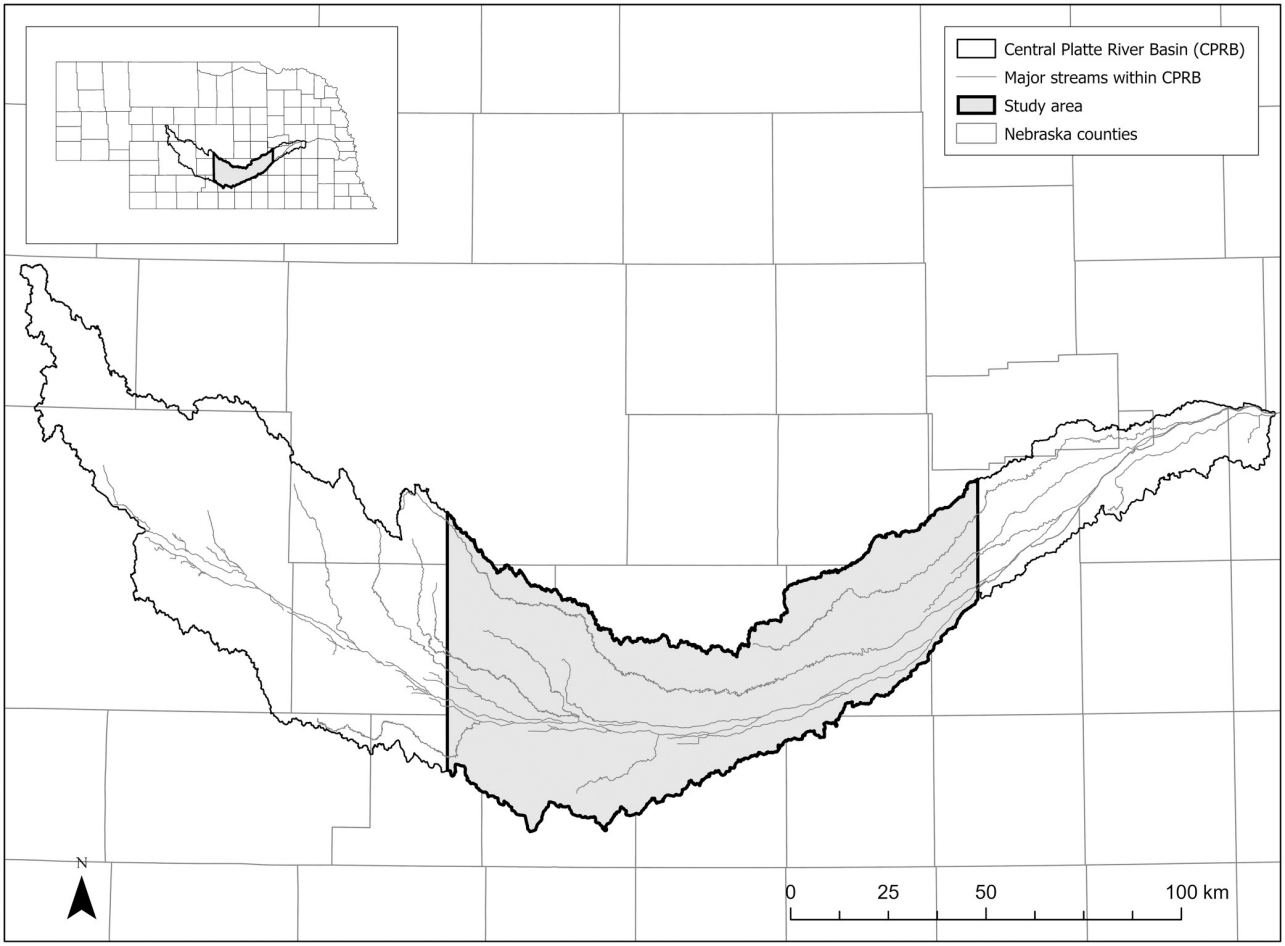
Study area encompassing the Big Bend Reach of the Central Platte River in central Nebraska, U.S.A. Figure visually represents spatial data from NebraskaMAP County Boundaries, HUC 8, and Major Streams datasets to show the location and hydrological features of the study area [37–39].

The Big Bend Reach is a 145-km stretch of the Central Platte River extending between Lexington, NE and Chapman, NE. Historically, the Big Bend Reach was a non-stationary ecological system in which the Central Platte River, a braided prairie stream, was shaped by periodic scouring flows [28, 29]. Following European settlement, the Big Bend Reach became more stationary due to the regulation of the river’s flow regime through damming and diversion and the management of the waters for purposes including irrigation and endangered species habitat [28]. Wetland land cover types, such as wet meadow and emergent marsh, are threatened by hydrological changes caused by the construction of the Kingsley Dam in 1941 and extensive diversion of water from the Central Platte River [30, 31]. Today, the area surrounding the Central Platte River is dominated by agriculture, specifically corn and soybean production [32]. Management in this more stationary system is challenged with providing habitat for endangered and other species while meeting human demands for irrigation and other water uses [33–35]. For example, the Big Bend Reach encompasses important habitat for mammal species of concern including the plains pocket mouse (*Perognathus flavescens*) and long-tailed weasel (*Mustela frenata*) [36]. Our study area (5,868 km^2^) encompassed the Platte River Basin, extending east and west to the bounds of the Big Bend Reach (Fig 1). Land cover data for the study area in raster format at 30-m resolution were provided by the Rainwater Basin Join Venture [32].

### Mammalian focal species

In accordance with previous applications of discontinuity theory seeking to examine ecosystem structure [e.g., 4, 22, 24], we compiled a list of all species of a single taxonomic group, mammals, in the CPRV. We used *Mammals of Nebraska* [40] and additional published sources to determine species ranges (S1 Table). Extirpated and extinct species (e.g., black bear [*Ursus americanus*]) previously present in the ecosystem were also included. Peripheral species, including species with ranges that have recently expanded into the study area but are still rare or transient (e.g., nine-banded armadillo [*Dasypus novemcinctus*]) and transient species, such as native species that have been recorded in the study area but are not known to have a breeding population, were not included. Body mass data were collected from published sources, primarily the *CRC Handbook of Mammalian Body Masses* (S1 Table) [41]. Data with the geographic proximity closest to the study area were selected for each species from the available body mass data. Male and female body mass data were averaged when both were available for a given species. If only male or female data were available, the data for the available sex were used.

### Discontinuity analysis

Applying discontinuity analysis to body mass distributions involves examining the differences between rank-ordered species body masses. Accordingly, we first ranked all mammalian focal species (n = 49) in ascending order of body mass. We analyzed the body mass distribution by comparing the distribution of the actual body mass data to a null distribution developed using a continuous unimodal kernel distribution of the log-transformed body mass data [42]. Discontinuities were identified as any gaps between successive species body masses that significantly exceeded the gaps created by the null distribution using a consistent statistical power. Species aggregations, or groups of species representing each scale in the system, were identified as any group of three or more successive species that were not separated by a discontinuity. We disregarded discontinuities that resulted in aggregations of fewer than three species [4].

### Mammal dispersal

Mammal species body mass is allometric to dispersal distance [27, 43]. We obtained dispersal data for the mammal species included in the body mass discontinuity analysis (S1 Table). For each mammal species, we selected natal or adult dispersal data from published sources using the following order of preference: measured as (1) the mean distance from the center or edge of the natal range to the center or edge of the adult home range; (2) the mean distance between recaptures, capture and death, or capture and loss of tracking; (3) the maximum distance from the center or edge of the natal range to the center or edge of the adult home range; (4) the maximum distance between recaptures, capture and death, or capture and loss of tracking; (5) based on home range size; (6) the cumulative distance moved over a given number of days; and (7) other dispersal measurements (e.g., long-distance movements between foraging sites, nightly movements, or movement between social groups). If multiple sources with similar methods were available for a given species, we selected data with the closest geographic proximity to the study area, with the largest sample size, or natal dispersal measurements. We selected dispersal measurements that were either for male and female individuals combined or, if unavailable, only for female individuals. Dispersal data were not available for some species. Although we utilized multiple types of dispersal measurements due to the limited availability of mammal dispersal data, the expected pattern of increasing dispersal distance with greater body mass size was present in the selected data [27, 43]. We converted all available dispersal distances to meters, then calculated the mean dispersal distance, rounded to the nearest hundredth, for the species in each aggregation in order to obtain a dispersal distance in meters representing every scale identified in discontinuity analysis.

### Evaluating connectivity

We applied a graph-theoretic network analysis approach to evaluate connectivity of the CPRV at multiple objectively identified scales [10, 12, 44, 45]. Using ArcGIS Pro 2.8.3 [46], we converted the 30-m resolution raster land cover data provided by the Rainwater Basin Joint Venture Nebraska Land Cover Development (2016 Edition) dataset to vector format and identified all patches of wet meadow and emergent marsh land cover in the study area [32]. To ensure that polygons sharing a common boundary at a vertex point were considered to be a single patch of habitat, we added a 0.01-m buffer to all polygons before using the Dissolve Boundaries tool to combine all patches sharing a common boundary. This small buffer ensured that the Dissolve Boundaries tool ran correctly but did not influence the connectivity analysis. We then calculated the total patch area, mean patch size, and number of patches of each land cover type in ArcGIS. Next, we used the Generate Near Table function to calculate the Euclidean edge-to-edge distances between all wet meadow and emergent marsh patches respectively at each scale. In other words, we identified all the patches of each land cover type within the scale-specific dispersal distance from each other. Notably, we selected these wetland land cover types to serve as an example application of our approach. Our focus was not on the connectivity of these land cover types for specific species, but instead on the relationship between scale and connectivity.

Using R 4.0.5 [47], we separately developed and analyzed networks for the wet meadow and emergent marsh land cover types at each scale using functions included in the packages tidyverse [48], igraph [49], and rgdal [50]. For each scale, the network was composed of nodes, which were patches of wet meadow or emergent marsh, and edges, which were the edge-to-edge connections between nodes within the given dispersal distance. We measured patch (i.e., node-level) connectivity using degree centrality, or the number of direct connections between a node and other adjacent nodes [13, 51]. A node with a high degree centrality represents a habitat patch that is within the given distance to many other patches of habitat, indicating that species can move from this patch to many other patches of habitat [45]. We evaluated landscape (i.e., network-level) connectivity using mean degree centrality, characteristics of network components, and modularity. Mean degree centrality is the mean number of edges adjoining each node in the network and describes the degree to which nodes in the network are connected to other neighboring nodes [13, 45]. A larger number of connections between neighboring nodes on average suggests a greater potential for species movement among patches of habitat in the network. We evaluated the characteristics of the components, or clusters of connected nodes, in each network by calculating the number of components in the network, the mean number of nodes in the largest component, and percent of nodes in the largest component [13, 45]. Patches of habitat are more disconnected for species moving through the landscape in a network consisting of many small, separate clusters of nodes, whereas a network consisting of fewer, larger clusters of nodes begets a more connected landscape for those species [13]. A highly connected network may consist of a single cluster of nodes, indicating that every habitat patch can be accessed directly or indirectly from all other patches in the network [13]. Modularity measures the extent to which there are highly connected subgroups of nodes with few connections between subgroups in the network [13, 52]. Although a high degree of modularity in a network may impede the movement of species between habitat patches, a moderate degree of both modularity and connectivity may facilitate movement while also limiting the negative effects of disturbances such as disease spread through the habitat network [53–55].

## Results

### Species and scale identification

**Fig 2:**
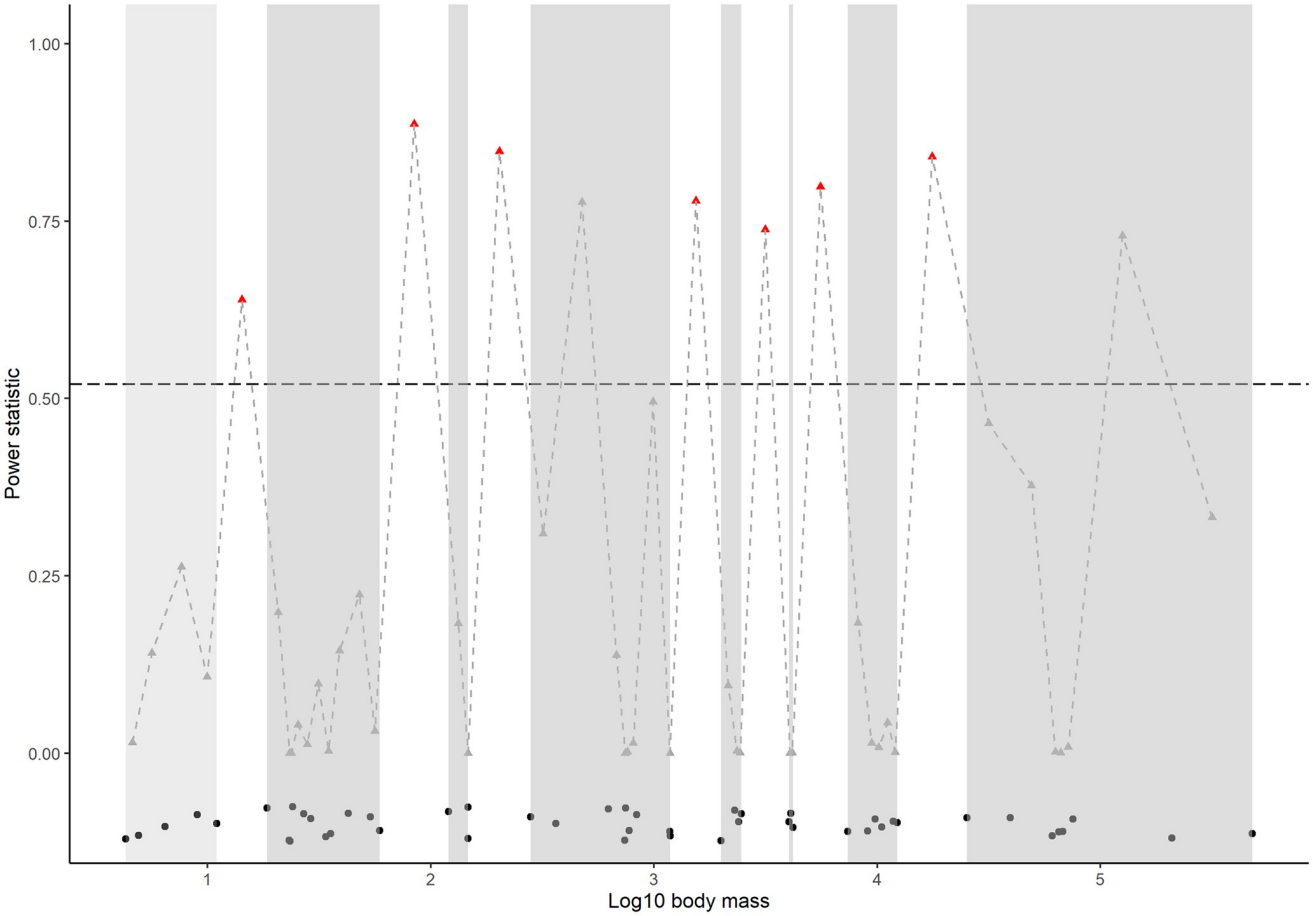
Discontinuities and mammal species aggregations in the Central Platte River Valley. The log_10_ body masses of all mammal species are represented by points (black) along the x-axis. The points are jittered for illustrative purposes. The power statistic (~0.50, n = 49) is shown by the slashed horizontal line (black). All gaps between species are represented by triangles; red triangles indicate discontinuities between species aggregations. Species aggregations (defined as groups of three or more species) are shaded (gray).

We identified 49 mammal species present or historically present in the CPRV study area (S1 Table). The body mass distribution of the mammal species was discontinuous. We identified eight aggregations of mammals in the data separated by seven discontinuities (Fig 2). The number of mammal species in each aggregation ranged from three species to eleven species. The average dispersal distance of mammal species in each aggregation increased with scale (Table 1). The longest mean dispersal distance was 67,500 m for mammal species at the largest scale, more than 300 times longer than the mean dispersal distance of 200 m for species at the smallest scale in the ecosystem.

**Table 1:** Mean dispersal distances for mammal body mass aggregations.

Species aggregation	Mean dispersal (m)
1–2	200
3	500
4	6500
5	8200
6	22700
7	27000
8	67500

Mean dispersal distances were rounded to the nearest hundredth. Due to the limited availability of mammal dispersal data and similarity of the mean dispersal distances for aggregations numbers one and two, those aggregations were combined as one scale for analysis.

### Network analysis

**Fig 3:**
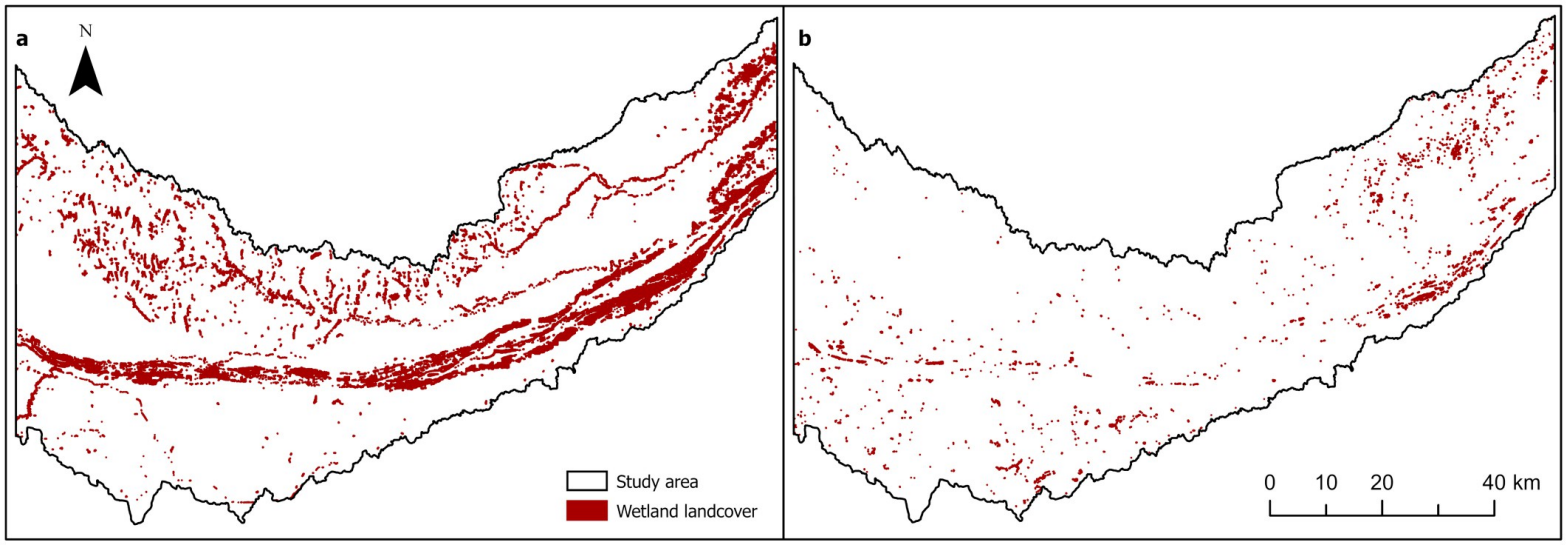
Map of (a) wet meadow and (b) emergent marsh land cover in the Central Platte River Valley study area. The study area included 6,330 patches of wet meadow land cover and 1,847 patches of emergent marsh land cover. Figure provides a visual representation of the spatial data from the NebraskaMAP HUC8 [38] dataset used to develop the study area and the Rainwater Basin Joint Venture Nebraska Land Cover Development (2016 Edition) [32] dataset used as land cover data in the analysis of landscape connectivity.

Our examination revealed that spatial characteristics of the two land cover types selected for our example analysis differed. The wet meadow land cover type presented a greater total area, greater mean patch area, and greater number of patches than the emergent marsh land cover type in the CPRV (Fig 3). The total area of wet meadow land cover in the study area was 208 km^2^, roughly 15 times larger than the area of emergent marsh land cover (14 km^2^). Similarly, the mean patch area for the wet meadow land cover type was 32,913 m^2^, approximately 4.5 times larger than the mean patch size of emergent marsh land cover (7,446 m^2^).

**Fig 4:**
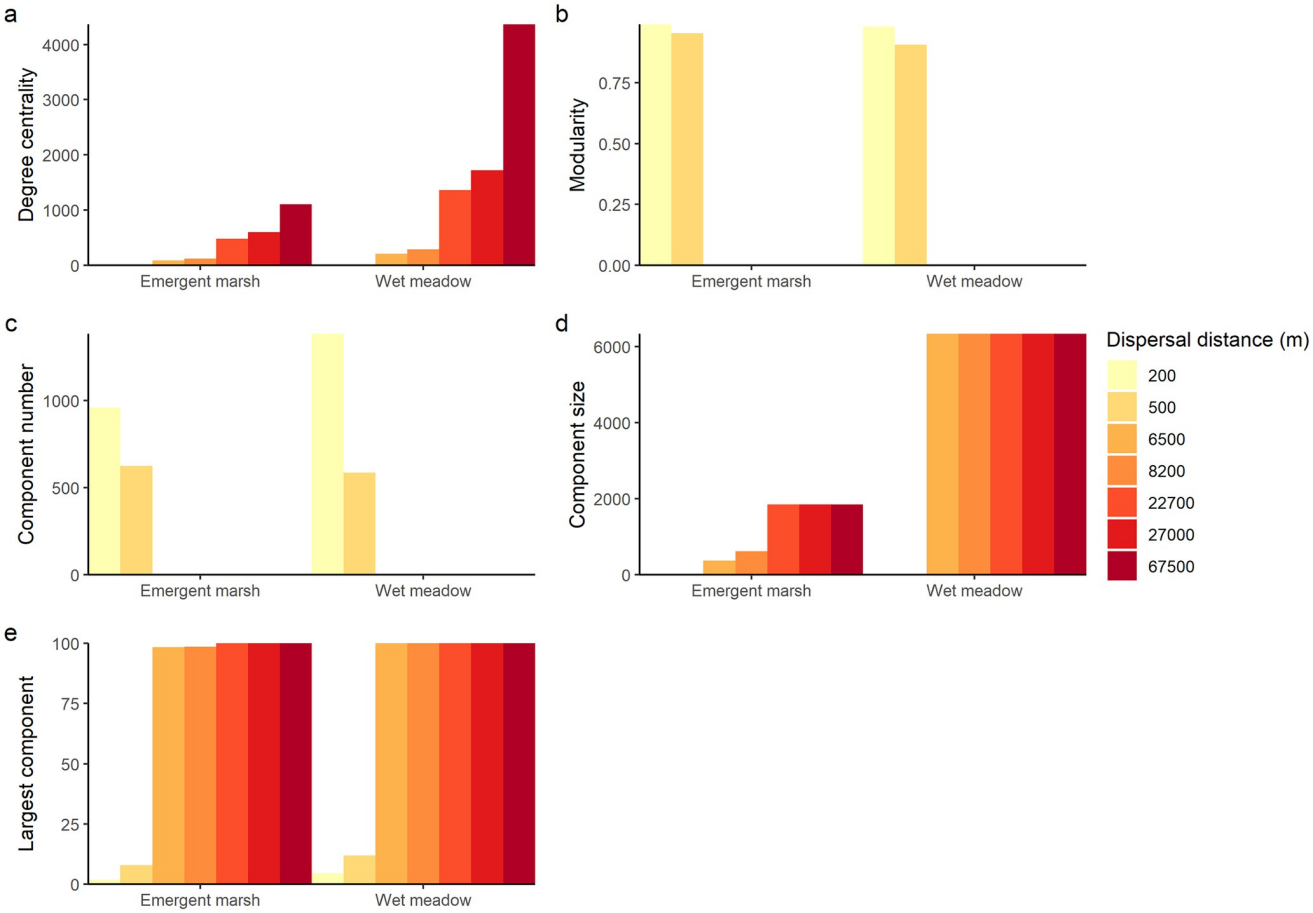
Evaluation of wetland connectivity in the Central Platte River Valley using seven dispersal distances. (a) Mean degree centrality, the mean number of direct connections each wetland patch has to other wetland patches. (b) Modularity, the strength of division in the wetland network. (c) Component number, the number of components of wetland patches. Components are groups of connected wetland patches. (d) Component size, the number of wetland patches in the largest component. (e) Largest component, the percentage of wetland patches in the largest component.

Landscape connectivity varied substantially by both land cover type and scale. For example, the mean degree centrality of the wet meadow network was greater than the mean degree centrality of the emergent marsh network at all scales (Fig 4). However, the wet meadow and emergent marsh networks demonstrated similar patterns of connectivity across scales in the landscape. As scale increased, mean degree centrality increased, modularity decreased, the number of components decreased, the mean component size increased, and the percent of nodes in the largest component increased (Fig 4).

For both wetland land cover types, a threshold of connectivity at which most nodes in the network were directly or indirectly connected to each other existed between the 500 m and 6,500 m dispersal distances of analysis. Between these dispersal distances, modularity decreased from 0.91 to zero in the wet meadow network and from 0.95 to zero in the emergent marsh network (Fig 4). Similarly, between the 500 m and 6,500 m distances, the percent of habitat nodes in the largest component increased from below 25% to over 95% for both wetland land cover types (Fig 4). For example, in the wet meadow network, 95% of nodes became present in the largest component at a dispersal distance of 2,250 m, whereas in the emergent marsh network, 95% of nodes became present in the largest component at a dispersal distance of 4,257 m.

Node-level connectivity followed the same pattern as the connectivity of the broader landscape. As scale increased, the number of isolated wetland patches, or patches with no connections to other wetland patches, decreased for both land cover types. For example, at the 200 m dispersal distance, there were 622 and 637 isolated patches and a maximum node degree of 12 and 31 in the emergent marsh and wet meadow networks, respectively (Table 2). At the 6,500 m dispersal distance, there were no isolated nodes in either network and the maximum node degree was 232 for the emergent marsh network and 459 for the wet meadow network, demonstrating that node-level connectivity increases with scale and supporting the connectivity threshold previously identified at the landscape level (Table 2).

**Table 2:** Maximum node degree and number of isolated nodes at seven dispersal distances.

Dispersal (m)	Maximum node degree		Number of isolated nodes	
	Wet meadow	Emergent marsh	Wet meadow	Emergent marsh
200	31	12	637	622
500	50	22	195	370
6500	459	232	0	0
8200	564	278	0	0
22700	1974	888	0	0
27000	2392	965	0	0
67500	5910	1713	0	0

## Discussion

Our results demonstrate the utility of body mass discontinuity analysis as a method to objectively identify scales in ecosystems for the evaluation of landscape connectivity at the level of ecological communities. In the CPRV, the body mass distribution of mammal species was discontinuous, indicating the presence of approximately eight scales in the ecosystem as utilized by mammals, each comprised of a unique set of mammal species, similar only in that they interact with their environment at a similar scale. Discontinuous body mass distributions have similarly been identified in animal communities in multiple ecosystems and for multiple taxa [4, 20, 22, 56–58]. The presence of aggregations of mammal species suggests that these groups of mammals interact with the landscape differently due to (1) movement capabilities that vary by species and especially by species size, represented in this analysis by dispersal distance; and (2) a scale-specific suite of structuring processes, disturbance responses, and habitat requirements [1, 5]. Applying body mass discontinuity analysis to identify scales in the context of landscape connectivity provides a method for landscape- and species-specific assessment of connectivity. This method may be especially helpful for addressing challenges of scale that emerge when evaluating connectivity for large numbers of species with varying dispersal distances. For example, mean dispersal distances among the eight mammalian body mass aggregations ranged from 200 to 67,500 m. If analyzed at 500 m increments similar to connectivity studies in neighboring regions [13], it would be necessary to compare 135 different scales of analysis, a task further complicated by the arbitrary selection of the 500 m increment size, which may still be too coarse to model landscape connectivity for some species such as the first two body mass aggregations identified in this study. The application of discontinuity theory with landscape connectivity assessments may be useful for targeting computationally intensive analyses to a relatively small, yet ecologically relevant, subset of scales, ultimately helping to avoid erroneous results and management recommendations. This analysis also demonstrates how the limited data requirements of body mass discontinuity analysis make this approach well-suited to identify ecosystem scales in situations with limited data availability or data collection capability [17]. Notably, we found that the availability of dispersal data for some mammal species, particularly small mammals, was limited. Additional research and data on animal movement would be valuable in improving our general understanding of animal responses to non-stationarity, as species with different dispersal capability and interacting with the landscape at different scales will respond differently to changes to the ecosystem and its structuring processes [1].

To provide an application of body mass discontinuity analysis in the context of landscape connectivity, we examined the general pattern of connectivity for mammals across scales in the CPRV. Previous studies of landscape connectivity in the CPRV area have selected a plausible range of scales for the analysis of connectivity [e.g., 13]. However, as landscape connectivity is highly dependent on the species present in a landscape, the use of discontinuity analysis provided a method to identify scales for the analysis of connectivity that is specific to the ecological community, in this case for mammal species, of a given landscape. Overall, we found that connectivity for mammal species varies substantially across scales in the CPRV. As scale increased, represented in this analysis by dispersal distance, connectivity of the landscape increased non-linearly. A threshold of connectivity existed between the 500 m and 6,500 m scales, and the landscape became highly connected for mammals at a dispersal distance of 2,250 m in the wet meadow network and 4,257 m in the emergent marsh network. At the threshold distance for both wetland types, the landscape shifted from being relatively unconnected with many isolated habitat patches to almost all habitat patches being directly or indirectly connected to each other. The presence of this threshold of connectivity suggests that human fragmentation of the landscape may primarily occur between the 500 m and 6,500 m scales in the CPRV, causing differing effects on landscape connectivity because species interact with the landscape at different scales [59]. For instance, a mammal species with a dispersal distance equal to or greater than the threshold dispersal distance can access almost all patches of wet meadow or emergent marsh land cover, respectively, in the landscape from a given patch of either wetland type. In contrast, those mammal species with dispersal distances below the threshold lack the ability to move easily between patches of wetland habitat in the fragmented landscape of the CPRV. Mammal species with relatively short dispersal distances (< 500 m) are likely confined to small groups of habitat patches, or components of the network, whereas species with longer dispersal distances (> 6,500 m) are able to move throughout the CPRV, which may affect these species’ vulnerability and responses to changes in land cover [13, 15, 60].

As such, this analysis of wet meadow and emergent marsh land cover types reveals patterns of landscape connectivity across scales in the CPRV that can be used to inform research and management efforts. The aggregations of mammal species identified in the discontinuity analysis will likely demonstrate scale-specific responses to habitat loss and habitat restoration, illustrating the importance of incorporating scale in management decisions. For example, mammal species with dispersal distances close to the connectivity threshold may be greatly affected by changes in habitat configuration because they rely on specific patches as stepping stones [6]. In contrast, mammals with relatively short or long dispersal capability may be less affected by changes in habitat configuration because the landscape remains largely unconnected or connected [6]. In this study area, species with dispersal distances above the connectivity threshold could likely directly or indirectly access many patches of wetland land cover in the landscape despite changes in habitat configuration.

Notably, this graph-theoretical analysis of landscape connectivity provides a systems-level examination of wetland connectivity for mammals in the CRPV and does not account for other factors in landscape connectivity such as landscape resistance. Future implementation of body mass discontinuity in the context of landscape connectivity could additionally include aspects of landscape permeability or implement approaches such as circuit theory that account for landscape resistance to provide more detailed assessment of connectivity [61]. As the results of this analysis are specific to mammals in the CPRV, the further application of discontinuity analysis to identify scales of interaction for birds or reptiles in the CPRV presents another potential opportunity to further develop the use of discontinuity analysis in the context of landscape connectivity.

Ultimately, management intended to enhance landscape connectivity must incorporate scale to ensure benefit to specific species or suites of species in the CPRV. If management is intended to increase or maintain the connectivity of the landscape for all mammal species, management for connectivity must occur at multiple scales, in particular at the scales around or below the connectivity threshold located between the 500 m and 6,500 m dispersal distances. Identification of a critical connectivity threshold suggests that in the absence of complete information, maintaining connectivity at a distance below the threshold will likely have the broadest benefit to species. Management for mammal species interacting with the landscape at greater scales and with longer dispersal distances may not benefit species at smaller scales due to their more limited ability to move among habitat patches. Discontinuity analysis provides a method to identify ecosystem structure, and the gaps and aggregations of species identified using this method will vary by system. Although previously dispersal distance has been thought to increase continuously, this analysis demonstrates that the scales of interaction of mammal species with the landscape increase discontinuously. The wide threshold of connectivity found in this analysis suggests that management with the assumption of continuously increasing dispersal distance could result in erroneous management actions. The lack of a multiscale, multispecies approach to management will likely restrict benefits of management to a subset of species that are present at the selected scale of management and neglect species at other scales, potentially eroding the resilience of the ecosystem [3].

## Supporting information

S1 Table Data for mammal species in the Central Platte River Valley. (PDF)
