# Supplementary material for: Functional connectivity varies across scales in a fragmented landscape
Source: PLoS One. 2023 Aug 9;18(8):e0289706. doi: 10.1371/journal.pone.0289706 (PMC10411743; doi:10.1371/journal.pone.0289706)
Supplement: S1 Table — (PDF) [file pone.0289706.s001.pdf]

S1 Table. Data for mammal species in the Central Platte River Valley.

Scientific name	Common name	Range source	Body mass (kg)	Body mass source	Dispersal distance (m)	Dispersal source
Cryptotis parva	North American least shrew	Genoways et al. (2008b)	0.0043	Silva & Downing (1995)		
Sorex cinereus	Masked shrew	Genoways et al. (2008b)	0.0049	Silva & Downing (1995)	260.5	Oleinichenko et al. (2020)
Reithrodontomys montanus	Plains harvest mouse	Genoways et al. (2008b)	0.00645	Geluso & Wright (2019)	67	Goertz (1963)
Perognathus flavescens	Plains pocket mouse	Genoways et al. (2008b)	0.009	Hazard (1982)	424.7[a]	French et al. (1968)
Reithrodontomys megalotis	Western harvest mouse	Genoways et al. (2008b)	0.011	Silva & Downing (1995)	67	Goertz (1963)
Peromyscus maniculatus	North American deer mouse	Genoways et al. (2008b)	0.0185	Silva & Downing (1995)	306.3	Rehmeier et al. (2004)
Zapus hudsonius	Meadow jumping mouse	Genoways et al. (2008b)	0.0232	Silva & Downing (1995)	362	Schorr (2003)
Blarina brevicauda	Northern short-tailed shrew	Jones & Findley (1954); Jones (1964); Genoways et al. (2008b)	0.02335	Silva & Downing (1995)	94.7	Faust et al. (1971)
Onychomys leucogaster	Northern grasshopper mouse	Genoways et al. (2008b)	0.024	Silva & Downing (1995)		
Peromyscus leucopus	White-footed deermouse	Genoways et al. (2008b)	0.0269	Silva & Downing (1995)	25	Jacquot & Vessey (1995)
Synaptomys cooperi	Southern bog lemming	Genoways et al. (2008b)	0.029	Silva & Downing (1995)		
Microtus ochrogaster	Prairie vole	Genoways et al. (2008b)	0.0339	Silva & Downing (1995)	28.7	McGuire et al. (1993)
Microtus pennsylvanicus	Meadow vole	Genoways et al. (2008b)	0.0356	Silva & Downing (1995)		

Chaetodipus hispidus	Hispid pocket mouse	Genoways et al. (2008b)	0.0427	Silva & Downing (1995)	424.7	French et al. (1968)
Mustela nivalis	Least weasel	Genoways et al. (2008b)	0.0535	Silva & Downing (1995)		
Dipodomys ordii	Ord's kangaroo rat	Genoways et al. (2008b)	0.059	Silva & Downing (1995)	100	Gummer (1997)
Scalopus aquaticus	Eastern mole	Genoways et al. (2008b)	0.12	Silva & Downing (1995)		
Ictidomys tridecemlineatus	Thirteen-lined ground squirrel	Genoways et al. (2008b)	0.1465	Silva & Downing (1995)	53.1	Rongstad (1965)
Mustela frenata	Long-tailed weasel	Genoways et al. (2008b)	0.147	Silva & Downing (1995)	1000	Erlinge (1977)
Geomys bursarius	Plains pocket gopher	Genoways et al. (2008a); Genoways et al. (2008b)	0.27975	Silva & Downing (1995)	378	Quinn et al. (2011)
Poliocitellus franklinii	Franklin's ground squirrel	Genoways et al. (2008b); Jones (1964)	0.363	Silva & Downing (1995)		
Spilogale putorius	Eastern spotted skunk	Genoways et al. (2008b)	0.624	Silva & Downing (1995)		
Mustela nigripes	Black-footed ferret	Jones (1964); Genoways et al. (2008b)	0.74	Silva & Downing (1995)		
Sciurus niger	Eastern fox squirrel	Genoways et al. (2008b)	0.748	Silva & Downing (1995)	3300	Wooding (1997)
Cynomys ludovicianus	Black-tailed prairie dog	Jones (1964); Genoways et al. (2008b)	0.776	Silva & Downing (1995)	2400	Garrett & Franklin (1988)
Neovison vison	American mink	Genoways et al. (2008b)	0.8355	Silva & Downing (1995)	26554.18	Mitchell (1961)
Ondatra zibethicus	Common muskrat	Genoways et al. (2008b)	1.175	Silva & Downing (1995)		
Sylvilagus floridanus	Eastern cottontail	Jones (1964); Genoways et al. (2008b)	1.185	Silva & Downing (1995)	41.06	Chapman & Trethewey (1972)

Mephitis mephitis	Striped skunk	Genoways et al. (2008b)	2	Silva & Downing (1995)	3000	Rosatte & Gunson (1984)
Lepus californicus	Black tailed jackrabbit	Genoways et al. (2008b)	2.3	Silva & Downing (1995)	11000	Smith et al. (2002)
Vulpes velox	Swift fox	Jones (1964); Genoways et al. (2008b)	2.4	Silva & Downing (1995)	13100	Nicholson et al. (2007)
Didelphis virginiana	Virginia opossum	Genoways et al. (2008b)	2.465	Silva & Downing (1995)	5700	Beasley & Rhodes (2012)
Vulpes vulpes	Red fox	Genoways et al. (2008b)	4.03	Silva & Downing (1995)	44800	Gosselink et al. (2010)
Marmota monax	Woodchuck	Genoways et al. (2008b); Forrester et al. (2019)	4.1	Silva & Downing (1995)	685	Swihart (1992)
Urocyon cinereoargenteus	Common gray fox	Jones (1964); Genoways et al. (2008b)	4.205	Silva & Downing (1995)		
Lontra canadensis	North American river otter	Jones (1964); Genoways et al. (2008b)	7.4	Silva & Downing (1995)	3950	Erickson & McCullough (1987)
Castor canadensis	American beaver	Genoways et al. (2008b)	9.07	Silva & Downing (1995)	10150	Sun et al. (2011)
Taxidea taxus	American badger	Genoways et al. (2008b)	9.81	Silva & Downing (1995)		
Lynx rufus	Bobcat	Genoways et al. (2008b)	10.5	Silva & Downing (1995)	57900	Hughes et al. (2019)
Canis latrans	Coyote	Genoways et al. (2008b)	11.8	Silva & Downing (1995)	53200	Hibler (1977)
Procyon lotor	Common raccoon	Genoways et al. (2008b)	12.3	Silva & Downing (1995)	9700	Gehrt & Fritzell (1998)
Canus lupus	Gray wolf	Jones (1964); Genoways et al. (2008b)	25.3	Silva & Downing (1995)	87700	Jimenez et al. (2017)
Antilocapra americana	Pronghorn	Jones (1964); Genoways et al. (2008b)	39.5	Silva & Downing (1995)	26300	Jacques & Jenks (2007)

Puma concolor	Mountain lion	Jones (1964); Genoways et al. (2008b)	60.9	Silva & Downing (1995)	67400	Newby et al. (2013)
Odocoileus hemionus	Mule deer	Genoways et al. (2008b)	65.133	Silva & Downing (1995)	22800	Skelton (2010)
Odcoileus virginianus	White-tailed deer	Genoways et al. (2008b)	68	Silva & Downing (1995)	41000	Nixon et al. (2007)
Ursus americanus	Black bear	Jones (1964); Pelton et al. (1999); Genoways et al. (2008b)	75.5	Silva & Downing (1995)	40000	Costello (2010)
Cervus canadensis	Elk	Jones (1964); Genoways et al. (2008b)	209.5	Silva & Downing (1995)	118000	Petersburg et al. (2000)
Bison bison	American bison	Jones (1964); Genoways et al. (2008b)	480	Silva & Downing (1995)	136850	Jung (2017)

[a]Dispersal distance is for the surrogate species long-tailed pocket mouse (*Chaetodipus formosus*).

References

Beasley, J., & Rhodes, O. (2012). Genetic structure of a Virginia opossum (*Didelphis virginia*) population inhabiting a fragmented agricultural ecosystem. *Canadian Journal of Zoology*, *90*(1), 101-109. https://doi.org/10.1139/z11-119

Chapman, J., & Trethewey, D. E. C. (1972). Movements within a population of introduced eastern cottontail rabbits. *Journal of Wildlife Management*, *36*(1), 155-158.

Costello, C. M. (2010). Estimates of dispersal and home-range fidelity in American black bears. *Journal of Mammalogy*, *91*(1), 116–121. https://doi.org/10.1644/09-MAMM-A-015R1.1

Erickson, D., & McCullough, C. (1987). Fates of translocated river otters in Missouri. *Wildlife Society Bulletin* (1973-2006), *15*(4), 511-517.

Erlinge, S. (1977). Spacing strategy in stoat *Mustela erminea*. *Oikos*, *28*(1), 32-42. https://doi.org/10.2307/3543320

Faust, B. F., Smith, M. H., & Wray, W. B. (1971). Distances moved by small mammals as an apparent function of grid size. *Acta Theriologica*, *16*, 161-177. https://doi.org/10.4098/AT.ARCH.71-11

Forrester, A. J., Peterson, B. C., Ringenberg, J. M., Schlater, S. M., & Geluso, K. (2019). Continued westward expansion of woodchucks (*Marmota monax*) in Nebraska. *Western North American Naturalist*, *79*(4), 574-580. https://doi.org/10.3398/064.079.0410

French, N. R., Tagami, T. Y., & Hayden, P. (1968). Dispersal in a population of desert rodents. *Journal of Mammology*, *49*(2), 272–280. https://doi.org/10.2307/1377984

Garrett, M., & Franklin, W. (1988). Behavioral ecology of dispersal in the black-tailed prairie dog. *Journal of Mammalogy*, *69*(2), 236-250. https://doi.org/10.2307/1381375

Gehrt, S., & Fritzell, E. (1998). Duration of familial bonds and dispersal patterns for raccoons in south Texas. *Journal of Mammalogy*, *79*(3), 859-872. https://doi.org/10.2307/1383094

Geluso, K., & Wright, G. D. (2019). Status of the plains harvest mouse (*Reithrodontomys montanus griseus*) in eastern Nebraska. *Transactions of the Nebraska Academy of Sciences*, *39*, 10–16. https://doi.org/10.32873/unl.dc.tnas.39.10

Genoways, H. H., Hamilton, M. J., Bell, D. M., Chambers, R. R., & Bradley, R. D. (2008a). Hybrid zones, genetic isolation, and systematics of pocket gophers (genus *Geomys*) in Nebraska. *Journal of Mammalogy*, *89*(4), 826–836. https://doi.org/10.1644/07-MAMM-A-408.1

Genoways, H. H., Hoffman, J. D., Freeman, P. W., Geluso, K., Benedict, R. A., & Huebschman, J. J. (2008b). Mammals of Nebraska. *Bulletin of the University of Nebraska State Museum*, *23*. https://museum.unl.edu/file_download/20c9453c-484d-43ed-a4e1-ab37fb11362d

Goertz, J. W. (1963). Some biological notes on the plains harvest mouse. *Proceedings of the Oklahoma Academy of Science*, *43*, 123-125.

Gosselink, T. E., Piccolo, K. A., van Deelen, T. R., Warner, R. E., & Mankin, P. C. (2010). Natal dispersal and philopatry of red foxes in urban and agricultural areas of Illinois. *Journal of Wildlife Management*, *74*(6), 1204-1217. https://doi.org/10.1111/j.1937-2817.2010.tb01241.x

Gummer, D. L. (1997). *Effects of latitude and long-term isolation on the ecology of northern Ord's kangaroo rats (Dipodomys ordii)* [Master's thesis, University of Calgary]. University of Calgary Legacy Theses. http://dx.doi.org/10.11575/PRISM/16704

Hazard, E. B. (1982). *The mammals of Minnesota*. University of Minnesota Press.

Hibler, S. J. (1977). *Coyote movement patterns with emphasis on home range characteristics* [Master's thesis, Utah State University]. Utah State University Repository. https://doi.org/10.26076/0b05-5381

Hughes, A., Reding, D., Tucker, S., Gosselink, T., & Clark, W. (2019). Dispersal of juvenile bobcats in a recolonizing population. *Journal of Wildlife Management*, *83*(8), 1711-1719. https://doi.org/10.1002/jwmg.21747

Jacques, C. N., & Jenks, J. A. (2007). Dispersal of yearling pronghorns in western South Dakota. *Journal of Wildlife Management*, *71*(1), 177-182. https://doi.org/10.2193/2005-704

Jacquot, J., & Vessey, S. (1995). Influence of the natal environment on dispersal of white-footed mice. *Behavioral Ecology and Sociobiology*, *37*, 407-412. https://doi.org/10.1007/BF00170588

Jimenez, M., Bangs, E., Boyd, E., Smith, D., Becker, S., Ausband, D., Woodruff, S., Bradley, L., Holyan, J., & Laudon, K. (2017). Wolf dispersal in the Rocky Mountains, Western United States: 1993-2008. *Journal of Wildlife Management*, *81*(4), 581-592. https://doi.org/10.1002/jwmg.21238

Jones, J. K., Jr., & Findley, J. S. (1954). Geographic distribution of the short-tailed shrew, *Blarina brevicauda*, in the Great Plains. *Transactions of the Kansas Academy of Science*, *57*(2), 208-211. https://doi.org/10.2307/3626023

Jones, J. K., Jr. (1964). Distribution and taxonomy of mammals of Nebraska. *University of Kansas Publications, Museum of Natural History*, *16*(1), 1-356.

Jung, T. (2017). Extralimital movements of reintroduced bison (*Bison bison*): Implications for potential range expansion and human-wildlife conflict. *European Journal of Wildlife Research*, *63*, 35. https://doi.org/10.1007/s10344-017-1094-5

McGuire, B., Getz, L., Hofmann, J., Pizzuto, T., & Frase, B. (1993). Natal dispersal and philopatry in prairie voles (*Microtus ochrogaster*) in relation to population density, season, and natal social environment. *Behavioral Ecology and Sociobiology*, *32*(5), 293-302. https://doi.org/10.1007/BF00183784

Mitchell, J. (1961). Mink movements and populations on a Montana river. *Journal of Wildlife Management*, *25*(1), 48-54. https://doi.org/10.2307/3796990

Newby, J. R, Mills, L. S., Ruth, T. K., Pletscher, D. H., Mitchell, M. S., Quigley, H. B., Murphy, K. M., & DeSimone, R. (2013). Human-caused mortality influences spatial population dynamics: Pumas in landscapes with varying mortality risks. *Biological Conservation*, *159*, 230-239. https://doi.org/10.1016/j.biocon.2012.10.018

Nicholson, K., Ballard, W., McGee, B., & Whitlaw, H. (2009). Dispersal and extraterritorial movements of swift foxes (*Vulpes velox*) in northwestern Texas. *Western North American Naturalist*, *67*(1), 102-108. http://dx.doi.org/10.3398/1527-0904(2007)67[102:DAEMOS]2.0.CO;2

Nixon, C., Mankin, P., Etter, D., Hansen, L., Brewer, P., Chelsvig, J., Esker, T. L., & Sullivan, J. (2007). White-tailed deer dispersal behavior in an agricultural environment. *The American Midland Naturalist*, *157*(1), 212-220. http://dx.doi.org/10.1674/0003-0031(2007)157[212:WDDBIA]2.0.CO;2

Oleinichenko, V. Yu., Raspopova, A. A., Meschersky, I. G., Kuptsov, A. V., Kalinin, A. A., Aleksandrov, D. Yu., Belokon, M. M., Belokon, Yu.S ., & Gritsyshin, V. A. (2020). Dispersal of young common shrews (*Sorex araneus*) from natal ranges. *Biology Bulletin*, *47*(9), 1214-1226. https://doi.org/10.1134/S1062359020090113

Pelton, M. R., Coley, A. B., Eason, T. H., Doan Martinez, D. L., Pederson, J. A., van Manem, F. T., & Weaver, K. M. (1999). American black bear conservation action plan (*Ursus americanus*). In C. Servheen, S. Herrero, B. Peyton (Eds.), *Bears: Status survey and conservation action plan* (pp 144-156). International Union for Conservation of Nature/Species Survival Commission, Bear Specialist Group, Polar Bear Specialist Group. https://www.researchgate.net/profile/Stephen-Herrero/publication/48376974_The_Status_Survey_and_Conservation_Action_Plan_Bears/links/00b49532c7f3d68039000000/The-Status-Survey-and-Conservation-Action-Plan-Bears.pdf

Petersburg, M., Alldredge, A., & De Vergie, W. (2000). Emigration and survival of a 2-year-old male elk in Northwestern Colorado. *Widlife Society Bulletin (1973-2006)*, *28*(3), 708-716. http://dx.doi.org/10.2307/3783623

Quinn, V., Tsai, C. C., & Zollner, P. (2010). Distribution of the plains pocket gopher (*Geomys bursarius*) in the grassland physiographic regions of Indiana. *Proceedings of the Indiana Academy of Science*, *119*(1), 87-94.

Rehmeier, R. L., Kaufman, G. A., & Kaufman, D. W. (2004). Long-distance movements of the deer mouse in tallgrass prairie. *Journal of Mammalogy*, *85*(3), 562-568. https://doi.org/10.1644/1383956

Rongstad, O. (1965). A life history study of thirteen-lined ground squirrels in southern Wisconsin. *Journal of Mammalogy*, *46*(1), 76-87. https://doi.org/10.2307/1377818

Rosatte, R. C., & Gunson, J. R. (1984). Dispersal and home range of striped skunks, *Mephitis mephitis*, in an area of population reduction in southern Alberta. *The Canadian Field Naturalist*, *98*(3), 315-319.

Schorr, R. A. (2003). *Meadow jumping mice (Zapus hudsonius preblei) on the U.S. Air Force Academy, El Paso County, Colorado: Populations, movement and habitat from 2000-2002.* Colorado Natural Heritage Program unpublished report to the Natural Resources Branch, U.S. Air Force Academy. https://mountainscholar.org/bitstream/handle/10217/47079/Meadow_Jump_Mice_ElPaso_2003.pdf?sequence=1&isAllowed=y

Skelton, N. C. (2010). *Migration, dispersal, and survival patterns of mule deer (Odocoileus hemionus) in a chronic wasting disease-endemic area of southern Saskatchewan* [Master's thesis, University of Saskatchewan]. University of Saskatchewan Repository. https://harvest.usask.ca/bitstream/handle/10388/etd-09172010-082126/Thesis_Nicole_Skelton.pdf?sequence=1&isAllowed=y

Smith, G., Stoddart, L., & Knowlton, F. (2002). Long-distance movements of black-tailed jackrabbits. *Journal of Wildlife Management*, *66*(2), 463-469. https://doi.org/10.2307/3803179

Sun, L., Müller-Schwarze, D., & Schulte, B. (2011). Dispersal pattern and effective population size of the beaver. *Canadian Journal of Zoology*, *78*(3), 393-398. http://dx.doi.org/10.1139/cjz-78-3-393

Swihart, R. K. (1992). Home-range attributes and spatial structure of woodchuck populations. *Journal of Mammalogy*, *73*(3), 604-618. https://doi.org/10.2307/1382032

Wooding, J. B. (1997). *Distribution and population ecology of the fox squirrel in Florida* [Master's thesis, University of Florida]. University of Florida Digital Collections. http://ufdcimages.uflib.ufl.edu/UF/00/09/73/74/00001/distributionpopu00woodrich.pdf
